# A Comparative Study of Paralympic Veterans with Either a Spinal Cord Injury or an Amputation: Implications for Personalized Nutritional Advice

**DOI:** 10.3390/jfmk10030305

**Published:** 2025-08-06

**Authors:** Ilaria Peluso, Anna Raguzzini, Elisabetta Toti, Gennaro Boccia, Roberto Ferrara, Diego Munzi, Paolo Riccardo Brustio, Alberto Rainoldi, Valentina Cavedon, Chiara Milanese, Tommaso Sciarra, Marco Bernardi

**Affiliations:** 1 Research Centre for Food and Nutrition (CREA-AN), 00178 Rome, Italy; 2 Department of Clinical and Biological Sciences, University of Turin, 10126 Turin, Italy; 3 Physical Medicine and Rehabilitation Unit, Italian Army Medical Hospital, 00184 Rome, Italy; 4 Joint Veteran Defence Center, Defense Institute of Biomedical Sciences, 00184 Rome, Italy; 5 Department of Medical Sciences, University of Turin, 10126 Turin, Italy; 6 Laboratory of Anthropometry and Body Composition, Department of Neurosciences, Biomedicine and Movement Sciences, University of Verona, 37129 Verona, Italy; valentina.cavedon@univr.it (V.C.); chiara.milanese@univr.it (C.M.); 7 Department of Physiology and Pharmacology “V. Erspamer”, Sapienza University of Rome, 00185 Rome, Italy

**Keywords:** nutrition, sport, disability, physical fitness

## Abstract

**Background**: Dietary advice for Paralympic athletes (PAs) with a spinal cord injury (PAs-SCI) requires particular attention and has been widely studied. However, currently, no particular attention has been addressed to nutritional guidelines for athletes with an amputation (PAs-AMP). This study aimed at filling up this gap, at least partially, and compared veteran PAs-SCI with PAs-AMP. **Methods:** A sample of 25 male PAs (12 with SCI and 13 with AMP), recruited during two training camps, was submitted to the following questionnaires: allergy questionnaire for athletes (AQUA), Nordic Musculoskeletal Questionnaire (NMQ), Starvation Symptom Inventory (SSI), neurogenic bowel dysfunction (NBD), orthorexia (ORTO-15/ORTO-7), alcohol use disorders identification test (AUDIT), and Mediterranean diet adherence (MDS). The PAs were also submitted to the following measurements: dietary Oxygen Radical Absorbance Capacity (ORAC) and intakes, body composition, handgrip strength (HGS), basal energy expenditure (BEE), peak oxygen uptake (VO_2peak_), peak power, peak heart rate (HR), post-exercise ketosis, and antioxidant response after a cardiopulmonary exercise test (CPET) to voluntary fatigue. **Results:** Compared to PAs-AMP, PAs-SCI had higher NBD and lower VO_2peak_ (*p* < 0.05), peak power, peak HR, peak lactate, phase angle (PhA) of the dominant leg (*p* < 0.05), and ORTO15 (*p* < 0.05). The latter was related to NBD (r = −0.453), MDS (r = −0.638), and ORAC (r = −0.529), whereas ORTO7 correlated with PhA of the dominant leg (r = 0.485). Significant differences between PAs-AMP and PAs-SCI were not found in the antioxidant response, glucose, and ketone levels after CPET, nor in dietary intake, AUDIT, AQUA, NMQ, SSI, BEE, HGS, and FM%. **Conclusions:** The present study showed that PAs-SCI and PAs-AMP display similar characteristics in relation to lifestyle, energy intake, basal energy expenditure, and metabolic response to CPET. Based on both the similarities with PAs-SCI and the consequences of the limb deficiency impairment, PAs-AMP and PAs-SCI require personalized nutritional advice.

## 1. Introduction

The International Paralympic Committee, the global governing body of the movement of the athletes with an impairment, organizes, every four years and “parallel” to the Olympic Games, some of the largest sportive events in the world named Summer and Winter Paralympic Games (PGs) [1]. In the last Summer PGs (Paris 2024), about 4400 athletes participated competing in 22 sports: Para archery, Para athletics, Blind 5-a-side soccer (for athletes with a visual impairment), Boccia, Para canoe, Para cycling, Para equestrian, Goalball, Para judo, Para powerlifting, Para rowing, Shooting Para sport, Sitting volleyball, Para fencing, Para swimming, Para table tennis, Para triathlon, Wheelchair basketball, Wheelchair rugby, Wheelchair tennis, Para badminton, and Para taekwondo. The latter substituted Parasailing and 7-a-side soccer (for athletes with cerebral palsy), which were present at the Paralympic level up to Rio 2016. In the Winter PGs, athletes compete in six sports: Para alpine skiing, Para biathlon, Para cross-country skiing, Para ice hockey, Para snowboard, and Wheelchair curling [1].

To be eligible to compete in Para sports, an athlete must have a diagnosis that leads to a permanent impairment [2]. This impairment must fall within one of the three main eligible impairment types: vision, intellectual, and physical/motor impairments [3]. The latter type includes eight impairments: impaired muscle power, impaired passive range of movement, limb deficiency, leg length difference, short stature, hypertonia, ataxia, and athetosis [2,3]. Physical impairments are divided into two subgroups: neurological and musculoskeletal impairments [3]. Among the Paralympic athletes (PAs), the most common health conditions that lead to neurological and musculoskeletal physical impairments are spinal cord injury (SCI) and amputation (AMP), respectively [3].

Apart from the cardiovascular-related physiopathology determined by the health conditions, several differences exist between PAs with SCI (PAs-SCI) and those with AMP (PAs-AMP). It is known for example that from a cardiovascular point of view, short- and long-term adaptations to sport/exercise occur in PAs-SCI [4], leading often to a reduced oxygen uptake peak (VO_2peak_), the measurement of cardiorespiratory fitness, which is inversely related to cardiovascular risk [5] and is higher in PAs-AMP compared to those with SCI [5]. During the Paralympic Games, PAs with neurological health conditions, such as SCI, lost more days per year due to infections and gastrointestinal problems compared with PAs with other health conditions [6].

On the other hand, regardless of the health condition, it has been suggested that pain occurs in wheelchair users because of incorrect posture, chronic overuse, and obesity [7]. The latter is a common condition in individuals with SCI [8] and AMP [9], and it is one of the most prevalent cardiometabolic risk factors [5,8,9]. Phase angle (PhA), measured by bioelectrical impedance analysis (BIA) and reflecting the extra- and intracellular water content/balance, has been suggested as a discriminator of sarcopenia in chronic musculoskeletal pain patients [10] and as a marker of oxidative stress [11].

Compared to healthy individuals, those with SCI have lower levels of exogenous antioxidants, but a similar antioxidant response to exercise [12]. In a previous study, we observed that post-exercise ketosis was associated with a reduced antioxidant response after a simulated wheelchair basketball match, with no differences in Wheelchair Basketball Athletes (WBAs) with different health conditions [13].

Although concerns about carbohydrate (CHO) intake and glycogen stores have been raised in athletes with SCI [14], limb deficiency determining muscle asymmetry should affect glycogen stores. Muscle asymmetry is related to pain in individuals with AMP [15]. It has been suggested that the Mediterranean diet (Med-D) [16,17] and plant-based diets [18,19] reduce musculoskeletal pain. Moreover, to reduce cardiometabolic risk, the Med-D has been suggested for individuals with SCI [20] and with lower limb amputation (LLA) [21]. A prospective intervention (calorie-restricted Med-D and circuit resistance training) in a cohort study involving 20 individuals with SCI reduced body mass index (BMI) and total fat mass, and improved glucose regulation, insulin sensitivity, and lipid profiles [22]. Additionally, resting energy expenditure (REE), fat oxidation, cardiorespiratory fitness, and dynamic strength increased [22].

Despite the potential of the Med-D and plant-based diets to reduce both the cardiovascular risk and musculoskeletal pain, the influence of the health conditions on digestive functions is among the determinants of the dietary behaviors of wheelchair users with both SCI and LLA [23].

It has been stated that energy balance, a fundamental health related parameter in PAs, is extremely difficult to be assessed [24] due to the measurements of both energy expenditure for the wide ranges of types, intensities, and durations of practiced sports [25,26] and activities of daily living [27] and energy intake for the physio-pathological characteristics of some PAs, such as those with SCI [28]. Moreover, high variability in resting metabolic rate exists in PAs, as well as dietary restrictions related to the health condition [28]. In particular, individuals with restrictions on eating due to gastrointestinal symptoms require an evaluation for orthorexia [29].

It is known that dietary advice for individuals and PAs-SCI requires particular attention [30,31,32], whereas an unresolved question is whether PAs-AMP should follow nutritional advice as able-bodied athletes. Indeed, at our best knowledge only two studies have specifically evaluated PAs-AMP [33,34]. In these papers, the PAs of both the Brazilian Wheelchair Women’s Basketball Team [33] and the male Brazilian Amputee Soccer Team [34] had lower CHO intake compared to the CHO recommendations for able-bodied athletes. On the other hand, Scaramella et al. [35] did not give conclusive recommendations for PAs-AMP. Indeed, from one point of view, they [35] suggested that appropriate CHO intake should be based on the lower end limit of the CHO range of able-bodied athletes for PAs with less active muscle mass (i.e., those with SCI or with double leg amputees) [35]. However, from another point of view, PAs-AMP should increase their CHO needs, because the possible inefficiency of movement of ambulant athletes with AMP may increase glycogen utilization [35].

This study aimed to deepen the knowledge on the appropriate nutritional regimen of PAs-AMP, comparing PAs-SCI with PAs-AMP using a multidimensional approach. To accomplish this aim, a descriptive cross-sectional observational study was conducted [36,37] with a mixed quantitative methodology (questionnaires, fitness and clinical measurements) [36] on veteran PAs of the “Gruppo Sportivo Paralimpico della Difesa” (GSPD) [38]. We hypothesize that, despite the expected differences in cardiorespiratory fitness, gastrointestinal symptoms, and eating behaviors, both PAs-SCI and PAs-AMP can have similar dietary habits and post-exercise glucose and ketone responses. Therefore PAs-AMP would necessitate a tailored nutrition plan [36,37] based on recommendations similar to those for PAs-SCI [14,30,31,32,35] rather than those for able-bodied athletes.

## 2. Materials and Methods

### 2.1. Recruitment, Study Design, and Characteristics of the Athletes

The study was conducted in accordance with the Declaration of Helsinki, and the protocol was approved by the Ethics Committee of the Italian Army Medical Hospital. All participants read and signed the informed consent form, and they knew that they could withdraw at any time. The presence of SCI or AMP was applied as inclusion criteria.

**Figure 1:**
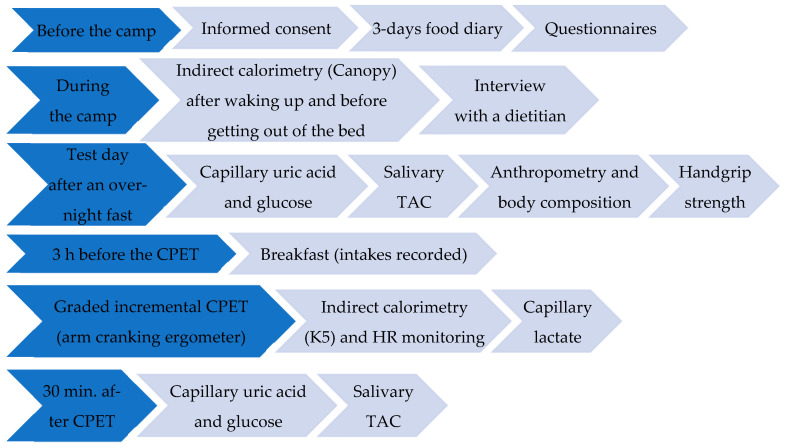
Schematic diagram of the study setting.

Recruitment was carried out among PAs participating in two training camps (May and September 2022, Jesolo, Italy) of the Italian Veterans. Thirty-nine eligible PAs (19 PAs-SCI and 20 PAs-AMP), participated in the two camps, among these 25 PAs (13 PAs-AMP and 12 PAs-SCI) signed the informed consent form to participate in the study. In this cross-sectional observational study, each PA followed the study setting described in Figure 1.

Data were collected through questionnaires (Figure 1), including the Starvation Symptom Inventory (SSI), neurogenic bowel dysfunction (NBD) score, and allergy questionnaire for athletes (AQUA); all of them have been previously used in wheelchair basketball players [39]. The health condition of each athlete was provided by the Joint Veteran Defence Center, Scientific Department, Army Medical Center, Rome, Italy. Wheelers used manual wheelchairs with propulsion-assist devices in their leisure time.

### 2.2. Anthropometric Measurements, Reported Pain, and Handgrip Strength

Body mass (BM, kg) was measured on an electronic wheelchair scale with an accuracy of 0.01 kg (Wunder RW 02, Trezzo sull’ Adda (MI), Italy). BM was calculated by subtracting the mass of the wheelchair and clothes (measured separately) from the total mass. Arm and waist circumferences were measured with a constant tension, non-elastic measuring tape meter (accuracy ± 1.0 mm). Arm span measurements were taken from the tip of the middle finger of one arm to the tip of the middle finger of the other arm with the arms outstretched at right angles of 180° to the body, with extended elbow and wrist, and the palms facing directly forward. Supine length was determined using a portable stadiometer (SECA 217, Gallarate, Italy, accuracy ± 0.5 mm). BMI was calculated (mass in kilograms divided by the square of height in meters) for PAs with bilateral LLA arm spam [40], and the amputation-adjusted BMI was calculated as previously described [41] with the calculator Amputee Coalition BMI Calculator Widget 2021 (amputee-coalition.org). After this normalization for amputation level, a BMI over 25 was considered the cut-off for overweight/obesity for PAs-AMP [42]. On the other hand, the “Guidelines for Identification and Management of Cardiometabolic Risk after Spinal Cord Injury” of the Consortium for Spinal Cord Medicine expert panel suggested a cutoff of BMI ≥ 22 kg/m^2^, given that waist circumference is not a validated proxy for obesity in individuals with SCI [43]. Therefore, we used this cut-off for the overweight/obesity prevalence evaluation in PAs-SCI. The Nordic Musculoskeletal Questionnaire (NMQ) [44] was administered to evaluate the reported pain. A dynamometer (DINAMOMETRO SMEDLEY—SA.NI.MEDICAL S.R.L., Rome, Italy) was used to measure the handgrip strength (HGS, as kilograms to the nearest 0.1 kg).

### 2.3. Body Composition and Phase Angle

Fat mass percentage (FM%) was calculated by 4 skinfolds (biceps, triceps, subscapular, and suprailiac ±0.2 mm) [45], measured with a skinfold caliper (Holtain Tanner/Whitehouse skinfold caliper 610 ND, Holtain Limited^®^, UK). Body density equations by Durnin and Womersley [46], converted to %FM using the Siri equation [47], were used. Phase angle (PhA) was measured using the BIA 101 BIVA^®^ PRO (Akern, Italy). The regional BIA assessment of single anatomical regions was performed using the tetrapolar technique that is useful to evaluate muscle asymmetries [48]. At the start of the measurement, participants were tested for at least 4 min in a supine position. For PAs-AMP, the electrodes were placed according to the literature [48].

### 2.4. Dietary Habits

Breakfast intake (buffet option, 3 h before the exercise test) was recorded on the day of the cardiopulmonary exercise test (CPET) (Figure 1). On the other hand, considering the circumstance/opportunity pattern [49] for dietary habits during a camp (full board) that could be a bias for the evaluation of differences in the habitual diet between study groups, questionnaires, food diaries, and interviews have been used to evaluate habitual dietary intake (Figure 1). In particular, dietary intake was evaluated through a 24 h food diary completed for 3 days prior to the camp (covering two consecutive weekdays and one weekend day) [50,51], followed by an interview with an expert dietitian to identify additional information considering the variation that is usually present in the diets [13,39]. The energy intake (EnI), the nutritional composition of the diet, and the dietary antioxidant capacity, expressed as Oxygen Radical Absorbance Capacity (ORAC), were calculated by the software Metadieta 3.0.1 (Meteda srl, Italy). Adherence to the Med-D was assessed with two different scores: the Mediterranean Diet Score (MDS, range 0–14: 14 items, each 0–1 score) and the Mediterranean Score (MEDScore, range 0–55: 11 items, each score range 0–5) [39]. The alcohol use disorders identification test (AUDIT) was used to assess alcohol-related problems (a score of 8 or more is considered to indicate hazardous or harmful alcohol use) [52]. The ORTO-15 and the ORTO-7, a shorter version of the ORTO-15 questionnaire, were used [53]. The ORTO-7 is based on items (1, 3, 4, 7, 9, 11 and 13) that mostly highlight the presence of orthorexia nervosa [53].

### 2.5. Basal and Maximal Oxygen Consumption

Basal oxygen uptake (VO_2_) and carbon dioxide production (VCO_2_) were measured following standard conditions (after waking up and before getting out of the bed) [54] by indirect calorimetry using a new generation of portable metabolimeter (Q-NRG plus, Cosmed, Italy) with a Canopy hood. After having reached the steady state conditions, measurements were continued for 10 min. Basal Respiratory Exchange Ratio (RER) and Basal Energy Expenditure (BEE) were therefore appropriately calculated (Omnia Software 2.2, Cosmed, Italy).

Two PAs with upper limb amputation (ULA, without prosthesis) and one with above-knee amputation (AKA, with pacemaker) did not perform the CPET. Therefore, 22 PAs (12 with SCI and 10 with AMP) carried out a CPET to volitional exhaustion to determine the VO_2peak_ [4]. The protocol consisted of a graded incremental (ramp) exercise test carried out with an arm cranking ergometer (ACE). The test started with a warm-up with no resistance (0 watt [W]) for 1 min and then continued with 2 min stages. The selected power for each stage depended on the health condition and the level of lesion of the athlete [26]. The protocols were designed to complete the test in about 10 min [45,55]. The cardiorespiratory measurements (heart rate—HR, pulmonary ventilation—VE, VO_2_, and VCO_2_) were obtained using the wearable K5 breath-by-breath metabolic cart (COSMED, Italy). The file was used to assess and quantify VO_2peak_, peak power, and HR_peak_ (Omnia Software, Cosmed, Italy).

### 2.6. Capillary Markers and Salivary Total Antioxidant Capacity

Before and after the CPET, lactate was measured from capillary blood samples (Figure 1) taken at the earlobe through a portable instrument (Lactate Pro2 LT-1730—Arkray, Japan) at rest, at the end of the CPET test, and during recovery. The multi-parametric Fora 6 (METER S.r.l., Italy) was used to evaluate capillary blood ketone, uric acid, and glucose concentrations, after an overnight fast (pre) and 30 min after the CPET (post) (Figure 1). At the same time periods (pre and 30 min. post CPET), the salivary Total Antioxidant Capacity (TAC) was measured (Figure 1) using the SAT test with the MiniSat instrument (H&D srl, Italy), according to the manufacturer’s instructions. Absolute changes in the concentrations of capillary markers and salivary TAC following the CPET were calculated as changes versus baseline concentrations.

### 2.7. Statistical Analysis

The significance of differences in categorical variables among groups was assessed by the Chi-square test, and the data were expressed as percentages. Continuous variables were expressed as means and standard errors of the means (SEMs) for results passing the normality test (Shapiro–Wilk test); otherwise, the data were expressed as medians (25–75% ranges). The *t*-test was used for normally distributed data, otherwise the Kruskal–Wallis test was performed. Spearman’s correlations (r = coefficient of correlation) were evaluated among variables. The level of significance was set at *p* < 0.05.

## 3. Results

### 3.1. Characteristics of the Athletes

Among the medal winners in Veteran National competitions in 2022 (seven PAs-AMP, five PAs-SCI and two with other health conditions), all but one participated in the study. A total of twenty medals were awarded in these Games by the recruited Veteran PAs across six disciplines, with nine gold, four silver, and seven bronze medals earned in Athletics, Swimming, Para-Badminton, Open Water Swimming, Archery, and Sailing. The prevalence of medalists in the two groups of PAs was: seven medalists out of thirteen recruited PAs-AMP and four medalists out of twelve recruited PAs-SCI.

The main characteristics of the PAs are described in Table 1.

All PAs-SCI and 23% of the PAs-AMP were wheelchair users (Table 1). No significant differences were found between groups in the time spent on different daily activities, as well as in sporting activities and in the number of body regions with reported pain (Table 1). However, 96% of the athletes reported pain in at least one part of the body. In both groups, the prevalence of shoulder pain (64% in PAs-AMP—71% in PAs-SCI) was above 60%, whereas the prevalence of pain in other body regions was below 50% (hip/thighs 45% in PAs-AMP and 14% in PAs-SCI, ankles/feet 27% in PAs-AMP and 14% in PAs-SCI, wrists/hands 18% in PAs-AMP and 29% in PAs-SCI). Differences among the percentages of athletes who reported trunk pain (27% of PAs-AMP and 57% of PAs-SCI) or neck pain (73% of PAs-AMP and 43% of PAs-SCI) did not reach statistical significance, whereas PAs-AMP had a higher (*p* < 0.05) prevalence of knee/s (54% of PAs-AMP—0% of PAs-SCI) and lumbar pain (83% of PAs-AMP—29% of PAs-SCI). Furthermore, a higher (*p* < 0.05) percentage of PAs-AMP used anti-inflammatory drugs (73%) compared to PAs-SCI (25%), while the use of analgesics was comparable (63.6% in PAs-AMP and 50.0% in PAs-SCI).

Half of the PAs-SCI had a neurogenic bladder (treatment with oxybutynin or solifenacin). Both the AQUA and SSI scores did not differ between groups, whereas the NBD score was obviously significantly higher in PAs-SCI compared to PAs-AMP (Table 1).

PAs-SCI had lower PhA values of the dominant leg than PAs-AMP, whereas no significant differences were observed in the PhA of the dominant arm, other anthropometric measures, and HGS (Table 2). Similar prevalences of overweight/obesity and smoking habits were found in PAs-SCI (75% and 14%) and PAs-AMP (69% and 10%).

### 3.2. Dietary Habits, Basal Metabolism, and Energy Intake

As described in Table 3, no significant differences were found in Med-D adherence and AUDIT scores. Median AUDIT scores (Table 3) were below those indicating hazardous or harmful alcohol use (cut-off of eight points for men) and none of the PAs-SCI or PAs-AMP showed hazardous alcohol use. ORTO-15 (but not ORTO-7) was significantly lower in PAs-SCI compared to PAs-AMP (Table 3). The prevalence of orthorexia was 9.1% and 33.3% in PAs-AMP and PAs-SCI, respectively. The normalized mean BEE expressed both as kcal/day or VO_2_/minute did not differ significantly between the two groups (Table 3). EnI and En% for all macronutrients, alcohol, and fiber were also similar in PAs-AMP and PAs-SCI (Table 3). ORAC and micronutrients of habitual diet did not differ significantly between groups (Table 4).

The day of the exercise test, PAs consumed the breakfast “ad libitum” (about 3 h before the CPET), with no differences between groups in EnI (327 ± 65 kcal in PAs-AMP vs. 347 ± 96 kcal in PAs-SCI), CHO %EnI (57 ± 7 in PAs-AMP, 49 ± 7 in PAs-SCI), fat %EnI (29 ± 5 in PAs-AMP, 35 ± 4 in PAs-SCI), dietary ORAC, and micronutrients (Table 4).

### 3.3. Response to the Cardiopulmonary Exercise Test (CPET)

VO_2peak_, HR_peak_, power, and peak lactate levels were lower in PAs-SCI compared to PAs-AMP (Table 5), whereas no significant differences were found in basal VO_2_ (Table 3).

Although two PAs-AMP had ketone levels above 0.6 mM, no significant differences were found in the variations in glucose (decrease) and ketone (increase) levels post CPET between groups (Table 5). Capillary uric acid levels and the salivary TAC increased after CPET with no differences between the two groups of PAs (Table 5).

### 3.4. Spearman’s Correlations

VO_2peak_ correlated with peak power (r = 0.834), peak HR (r = 0.783), peak lactate level (r = 0.472), BEE (r = 0.491), and PhA of the dominant leg (r = 0.587). Additionally, peak power correlated with HGS (r = 0.580) and PhA of the dominant arm (r = 0.419). The latter correlated with EnI (r = 0.636), protein intake (r = 0.636), and ORAC (r = 0.591).

On the other hand, capillary uric acid levels correlated with the salivary TAC (r = 0.575). Post-exercise ketosis was inversely related to both CHO %EnI (r = −0.530) and the increase in uric acid levels (r = −0.516, *p* < 0.05), whereas it positively correlated with SSI (r = 0.423).

Fat %EnI was inversely related to VO_2peak_ (r = −0.503), peak power (r = −0.665), PhA of the dominant arm (r = −0.482), Med-D adherence (MEDscore r = −0.817, MDS r = −0.626) and fiber intake (r = −0.532).

The ORTO15 and the ORTO7 (reverse scores: high values indicate low orthorexia) were related to both EnI and PhA of the dominant arm (Table 6). ORTO15 was related to NBD, MDS, ORAC, proteins in g/kg, and fiber intakes (Table 6). Moreover, higher values of ORTO15 (less orthorexia) correlated with FM%, BMI, and fat %EnI (Table 6).

## 4. Discussion

The multidimensional evaluation through questionnaires and clinical and functional measurements carried out in the present study in veteran athletes with physical (motor) impairments determined by different health conditions (SCI and AMP) showed a wide range of responses and a great overlap of results. Although the PAs in the present study presented the expected differences in cardiorespiratory fitness (lower in PAs-SCI than PAs-AMP) and gastrointestinal symptoms (NBD scores higher in PAs-SCI than PAs-AMP), adherence to the Med-D, lifestyle characteristics (sedentary behavior and sleeping time), and the number of painful sites were not different. Moreover, similar values were found for the nutritional status and dietary intake, basal metabolism, and glucose and ketone levels after CPET, and body composition in the parts of the body without impairment. Therefore, we verified the hypothesis that athletes with an impairment need a tailored nutrition plan based on a comprehensive clinical and functional assessment that also includes and evaluation of eating behavior (e.g., eating habits, preferences, and orthorexia).

The Paralyzed Veterans of America (PVA) dietary criteria include a nutrition plan similar to the Med-D (high levels of whole grains, fruits, vegetables, legumes, and low-fat dairy products, and low levels of red meat and sugar) [56]. In the present study, adherence to the Med-D was equal to about 50%, comparable to that observed in international-level WBAs [39] but with lower values of dietary ORAC, similar to those reported in obese individuals with low adherence to the Med-D [57] and definitively lower than those found in professional cyclists during training [58]. A significant negative correlation between the Med-D adherence and the ORTO-15 score has been previously reported in both professional (r = −0.365) and recreational (r = −0.309) able-bodied athletes [59]. Accordingly, the already quoted previous study of ours revealed through Spearman’s correlation data on WBAs indicated that WBAs with a low ORTO-15 score (high orthorexia) had a high MEDScore (r = −0.539) [39]. In the present study, we confirmed that Spearman’s correlations showed that PAs with high orthorexia, assessed through ORTO-15, had high adherence to the Med-D. ORTO-15 was also related to NBD scores and ORAC. It has been suggested that ORTO-15 may be related to a commitment to wellness in WBAs [39]. Contrary to ORTO-15, which can reveal positive healthy characteristics, in university students, the ORTO-7 was more specific than ORTO-15 in assessing orthorexia nervosa, being independent from confounding variables such as body image concerns, distress, appearance, fitness, and health orientation [53]. In our study, both ORTO-15 and ORTO-7 inversely correlated with EnI (ORTO-15 r = −0.542, ORTO-7 r = −0.624) and PhA of the dominant arm (ORTO-15 r = −0.579; ORTO-7 r = −0.500). Using ORTO-7, the negative correlation between ORTO-15 score and Med-D adherence was not found. Accordingly, Med-D adherence was not related to ORTO-7 in gym attendees with an FM% below 17 [39]. In the present study, the differences between groups in FM% did not reach statistical significance, but the FM% of PAs-SCI was found to be higher than previously reported data for PAs-SCI [60]. On the other hand, the FM% of PAs-AMP was comparable to previously reported data found in PAs with above-knee amputation (AKA) or below-knee amputation (BKA) [61]. We must stress the fact that not only the PAs-SCI but also the PAs-AMP displayed FM% higher than alpine and Nordic skiers and Para ice hockey players [45]. Indeed, the PAs in the present study, regardless of the health conditions, had an FM% similar to the Para Curlers who had an FM% equal to 26.2 ± 7.74% [45].

A recent position statement based on expert consensus suggests that the health and fitness evaluation of the preparticipation screening should include nutritional and body composition assessments and a daily energy expenditure (DEE) evaluation [24]. The energy expenditure of individuals with a locomotor impairment in both physical exercise and Paralympic sports vary widely depending on the type of physical activity, the actual intensity, and the duration of both exercise and sports [25,26]. Mean energy expenditure, for example, in sitting intermittent sports (aerobic and anaerobic mixed metabolism), such as wheelchair fencing, wheelchair tennis and wheelchair basketball, and endurance sports, such as sitting Nordic skiing and long-distance wheelchair racing, ranges between 7.21 and 11.7 metabolic equivalents of tasks (Mets), respectively [25]. The assessment of DEE becomes even more difficult during sport training in mixed metabolism sports, such as wheelchair basketball (circumstance sports) and during technical training. A DEE assessment in alpine sports training can also vary widely. Furthermore, the assessment of DEE is also dependent on the activities of daily living, which are difficult to assess [27]. Due to the difficulty in the evaluation of energy balance [24], a strict periodic evaluation should be carried out, addressing the point of tailored nutrition advice.

In the present study, the fat %EnI was higher than the reported one in high-performance PAs [62], whereas the observed low intake of CHO is consistent with the previous literature related to other PAs [13,63,64]. In a review on CHO consideration for PAs, it has been pointed out that the CHO oxidation rate was lower in PAs-SCI compared to able-bodied athletes, and that they have a greater dependency on CHO timing, due to the reduced glycogen storage capacity [14]. Accordingly, we proposed that elite WBAs with relatively low CHO intake could be at risk of both malnutrition and post-exercise ketosis [13]. Furthermore, it has been suggested that able-bodied individuals, following a session of aerobic exercise, presented increased TAC and uric acid levels as a protective reaction against oxidative stress [65]. In the present study, after CPET, both salivary TAC and blood uric acid levels increased, with no differences between PAs-AMP and PAs-SCI in the antioxidant response to CPET, as well as in glucose and ketone levels after CPET.

Positive correlations between TAC and PhA have been observed in patients with chronic kidney disease and older women [11]. In individuals with chronic musculoskeletal pain, the proposed cut-off value for PhA discriminating sarcopenia was 5.1 for men [10]. In the present study, the mean PhA value was below the cut-off for sarcopenia [10] only in the lower limbs of PAs-SCI.

On the other hand, the mean intake of proteins normalized for BM (mean values ranging from 1.2 to 1.4 g/kg BM) was adequate for PAs (1.2–1.7 g/kg BM) [20]. Although a high-protein intake diet might have a negative effect on kidney function in individuals with SCI, malnutrition can increase the risk of pressure ulcers [32]. The latter increases protein needs in sedentary individuals with SCI (at least 1.25 g/kg BM) for wound healing [32]. Furthermore, because stump ulcers are common problems in amputees using prosthetic limbs [66], a diet rich in protein would be advisable.

In veterans with unilateral BKA (97.9% with a prosthetic limb), a high prevalence of pain was reported (stump pain 84.2%, low back pain 78.1%, and knee pain 54.7%) and low back pain was higher in amputees with stump pain [67]. Comparing reported pain, we observed a high prevalence of both lumbar pain and use of anti-inflammatory drugs in PAs-AMP compared to PAs-SCI, whereas the use of analgesic drugs was comparable. Chronic pain is common (80%) in individuals with SCI [68] and it is known that neuropathic pain in both lower trunk and legs and spasticity can be observed in individuals with SCI [69]. In a previous study, the prevalence of neuropathic pain was 51% and 38% of patients with cervical and thoracic SCI, respectively, and 85% of patients with thoracic SCI and 87% of those with cervical SCI experienced spasticity [69]. Overall, in our study the prevalence of musculoskeletal pain at any location was higher (96%) than that previously reported in wheelchair users (50%) [7]. In both groups, we found the well-documented high prevalence of shoulder pain in wheelchair athletes (68%) and nonathletic wheelchair users (67%) [70]. Although it has been suggested that shoulder pain has a significant impact on the range of motion, leading to functional limitations [7], the absence of relationship between number of regions with reported pain and VO_2peak_ suggested that pain did not affect the execution of CPET on the arm cranking ergometer.

Health conditions, the type of impairment, and level of lesion impact VO_2peak_, and therefore the central (heart) and peripheral (vascular and muscular systems) short- [4] and long-term [71] adaptations to exercise differ when comparing PAs-AMP and PAs-SCI [4,71]. For this reason, VO_2peak_, which has an impact on performance in endurance, power, and intermittent (aerobic-anaerobic alternated) sports [45], shows also an inverse relationship with cardiovascular risk factors [5]. VO_2peak_ is therefore a fundamental component of physical fitness to be assessed in PAs competing in different sports [72]. In our study, cardiorespiratory fitness (VO_2peak_ and peak power) was higher in PAs-AMP compared to PAs-SCI. PAs-AMP had VO_2peak_ values consistent with the values of the Italian National WBAs [13,26]. Because the intensity and energy expenditure of a sport discipline has a strict relationship with VO_2peak_ [26], PAs competing in different sports display different VO_2peak_ values [26,45,72]. Therefore, the major limitation of our study is that the limited sample size prevented us from pooling athletes in sport discipline groups.

The first point of strength of the present paper is that we collected parameters with non-invasive (VO_2_, HR, and saliva) or minimally invasive (capillary blood) methods, making the study easily transferable on a large-scale during training camps. The second point of strength is that performing the test during training camps allowed us to measure BEE after waking up and before getting out of bed by indirect calorimetry, according to the best practice suggested in the literature [30,54]. In the present study, differences in both BEE and FM% between groups did not reach significance, suggesting that limb muscle asymmetry due to amputation generates similar effects to those observed in PAs-SCI with different upper and lower limb PhA. However, the limited sample size prevented us from subgrouping athletes for type and level of injury (i.e., PAs with AKA–BKA, unilateral–bilateral AMP, and paraplegia versus tetraplegia). Moreover, the selected population recruited, veteran PAs from the GSPD [30], could reduce the generalization of the results to other PAs. From that, further studies are needed to give specific and definitive recommendations.

## 5. Conclusions

Nutrient requirements for PAs are influenced by numerous intrinsic (health condition, impairment, and body composition) and extrinsic factors (lifestyle and type and intensity of the practiced sport). The present experimental study, aimed at clarifying the appropriate methodology to prescribe the best diet for PAs-AMP, utilizes fundamental and typically used measurements and questionnaires and introduces new (for PAs) measurements, such as metabolic responses to CPET and lifestyle questions. The results of the study showed substantial similarities between PAs-SCI and PAs-AMP, but with a wide range of responses. Indeed, reported pain, starvation symptoms, as well as antioxidant and glucose responses to CPET did not differ between the two groups of PAs. Therefore, the nutritional consideration for CHO intake due to the high risk of diabetes in PAs-SCI [5,14] should be made also for PAs-AMP, also considering that PAs with LLA showed higher blood GLU levels compared to PAs-SCI in our previous study [73]. Furthermore, Med-D adherence and dietary intakes of the PAs with different health conditions were also comparable, despite NBD and ORTO-15 scores being significantly different between groups. A high prevalence of orthorexia nervosa has been found in able-bodied individuals focused on sports performance or body composition (34.5%) [74], whereas in PAs it was related to gastrointestinal symptoms [39]. These data demonstrate that PAs with a locomotor impairment are, in some ways, fragile individuals, despite being athletes, and therefore they should be also evaluated from a clinical point of view using also laboratory measurements. In conclusion, confirming our hypothesis (PAs-AMP should follow the nutritional advice for PAs-SCI rather than those typical of able-bodied athletes), both PAs-SCI and PAs-AMP require detailed clinical and functional evaluations and assessments to facilitate the development of personalized nutritional advice. From this, the “single case experimental design” recently suggested for PAs in each feature of the clinical and sport life [75] could be the best practice to manage nutrition in Paralympians with a motor impairment.

## Figures and Tables

**Table 1:** Characteristics of the athletes.

	PAs-AMP	PAs-SCI
Age, years	45.2 ± 3.0	45.2 ± 3.4
	4 AKA (prosthesis, n = 1/4)	3 with tetraplegia (C6-C7)
	6 BKA (prosthesis, n = 5/6)	9 with paraplegia (T3-T12)
	3 ULA (prosthesis, n = 1/3)	
	Wheelchair users (3/13)	Wheelchair users (12/12)
Time since injury, years	15.7 ± 3.3	14.7 ± 3.3
Pain, n regions	3.9 ± 0.6	2.6 ± 0.8
Years of sport practice	8.2 ± 1.7	8.0 ± 1.6
Practiced sports	Athletics, Basketball, Cycling, Sitting volleyball, Swimming, Wheelchair Tennis.	Athletics, Archery, Hand-bike, Sitting volleyball, Swimming, Power Soccer.
Sport training, h/week	5.5 ± 0.7	5.9 ± 0.8
Television, h/week	10.8 ± 3.3	11.4 ± 2.3
Screening, h/week	23.3 ± 7.4	19.4 ± 4.1
Sleeping, h/day	7.0 (6.0 to 8.0)	8.0 (7.2 to 8.0)
AQUA	1.5 (0.0 to 10.0)	2.0 (0.5 to 7.2)
SSI	9.1 ± 2.0	7.0 ± 1.9
NBD	0.0 (0.0 to 0.0)	8.0 (4.5 to 11.7) *

PAs-AMP, Paralympic athletes with amputation; PAs-SCI, Paralympic athletes with a spinal cord injury; AKA, above-knee amputation; BKA, below-knee amputation; ULA, upper limb amputation; C, cervical; T, thoracic; AQUA, allergy questionnaire for athletes; SSI, Starvation Symptoms Inventory; NBD, neurogenic bowel dysfunction; * *p* < 0.05.

**Table 2:** Anthropometry and handgrip strength.

	PAs-AMP	PAs-SCI
BM, kg	81.9 (76.0 to 86.5)	83.0 (72.1 to 86.1)
Height, cm	179.1 ± 2.6	180.2 ± 2.3
Arm spam, cm	186.2 ± 2.4	181.8 ± 2.4
Arm circumference, cm	34.8 ± 1.2	33.7 ± 1.0
Waist circumference, cm	93.8 ± 4.3	98.9 ± 5.9
BMI, kg/m^2^	26.6 (24.1 to 28.8)	24.8 (22.3 to 26.4)
FM%	25.3 ± 2.5	28.8 ± 2.8
PhA dominant leg	6.7 ± 0.6	3.9 ± 0.4 *
PhA dominant arm	7.9 ± 0.5	7.7 ± 0.4
HGS, kg	42.3 ± 2.9	33.8 ± 4.6

PAs-AMP, Paralympic athletes with amputation; PAs-SCI, Paralympic athletes with a spinal cord injury; BM, body mass; BMI, body mass index; FM, fat mass; PhA, phase angle. HGS, handgrip strength. * *p* < 0.05.

**Table 3:** Dietary habits, basal metabolism, and energy intake.

	PAs-AMP	PAs-SCI
MDS, %	43.6 ± 3.1	42.9 ± 5.8
MEDScore, %	61.6 ± 2.3	57.7 ± 2.2
AUDIT	2.9 ± 0.4	4.2 ± 0.5
ORTO-15	37.8 ± 0.9	34.3 ± 1.0 *
ORTO-7	20.3 ± 0.7	18.8 ± 0.6
BEE, kcal/kg BM/d	23.1 ± 1.7	19.7 ± 1.0
EnI kcal/kg BM/d	27.1 ± 2.6	27.6 ± 2.8
Proteins, g/kg BM/d	1.4 ± 0.2	1.2 ± 0.1
Fat, %EnI	31.6 ± 1.9	35.7 ± 2.2
CHO, % EnI	44.8 ± 1.6	43.5 ± 1.3
Alcohol, % EnI	0.4 (0.2 to 2.9)	0.9 (0.0 to 2.8)
Fiber, % EnI	2.0 ± 0.3	1.8 ± 0.2
Fiber, g/1000 kcal	10.5 ± 1.5	9.4 ± 1.0
Fiber, g/d	21.1 ± 3.0	18.0 ± 1.8
Basal-RER	0.8 ± 0.1	0.8 ± 0.1
Basal VO_2_, mL/kg BM/min	3.4 ± 0.3	2.9 ± 0.1

PAs-AMP, Paralympic athletes with amputation; PAs-SCI, Paralympic athletes with a spinal cord injury; MDS, Mediterranean diet score; MEDScore, Mediterranean score; AUDIT, alcohol use disorders identification test; ORTO, score for orthorexia; BEE, basal energy expenditure, BM, body mass; EnI, energy intake; macronutrient, fiber, and alcohol intake as percentages of total energy intake (%EnI); CHO, carbohydrates; RER, Respiratory Exchange Ratio; VO_2_, oxygen uptake. * *p* < 0.05.

**Table 4:** Dietary antioxidant capacity and intake of micronutrients.

	PAs-AMP—Diet	PAs-AMP—Breakfast	PAs-SCI—Diet	PAs-SCI—Breakfast
Dietary ORAC, mM	6.5 ± 1.6	0.0 (0.0 to 0.8)	6.6 ± 1.6	0.0 (0.0 to 0.4)
Beta-carotene, mcg	2614.5 ± 550.2	18.3 (4.5 to 32.0)	2346.7 ± 483.0	26.0 (0.0 to 32.0)
Vitamin A, mcg	925.1 ± 155.4	29.8 (3.7 to 66.5)	1035.9 ± 237.4	41.1 (0.0 to 168.0)
Vitamin B1, mg	1.0 ± 0.1	0.2 ± 0.1	0.9 ± 0.2	0.2 ± 0.1
Vitamin B2, mg	1.6 ± 0.1	0.2 ± 0.1	1.3 ± 0.2	0.3 ± 0.1
Vitamin B3, mg	26.0 ± 2.2	2.7 ± 0.5	20.5 ± 2.8	3.3 ± 0.5
Vitamin B5, mg	2.5 ± 0.3	0.3 ± 0.1	2.6 ± 0.3	0.3 ± 0.1
Vitamin B6, mg	2.0 ± 0.2	0.1 ± 0.1	1.6 ± 0.2	0.1 ± 0.1
Vitamin B9, mcg	279.7 ± 25.9	21.2 ± 4.6	263.2 ± 43.6	19.4 ± 5.9
Vitamin B8, mg	21.6 ± 3.1	1.4 (1.0 to 3.7)	21.4 ± 3.2	1.4 (1.2 to 3.6)
Vitamin B12, mcg	5.1 (3.8 to 8.7)	0.0 (0.0 to 0.4)	4.6 (2.7 to 6.8)	0.1 (0.0 to 0.7)
Vitamin C, mg	97.6 ± 14.5	0.7 (0.1 to 15.0)	117.9 ± 28.5	0.0 (0.0 to 2.1)
Vitamin D, mcg	1.3 (1.1 to 3.1)	0.1 (0.0 to 0.2)	2.7 (1.3 to 3.1)	0.1 (0.0 to 0.2)
Vitamin E, mg	8.7 ± 1.5	0.2 (0.1 to 0.3)	8.6 ± 1.8	0.1 (0.0 to 0.3)
Vitamin K, mcg	1.6 (0.26 to 4.7)	0.1 ± 0.1	0.7 (0.1 to 4.7)	0.0 ± 0.0
Calcium, mg	1072.4 ± 129.2	179.8 ± 83.9	958.9 ± 138.9	222.6 ± 79.5
Phosphorus, mg	1341.4 ± 71.9	163.7 ± 60.4	1088.6 ± 80.6	204.5 ± 67.0
Sodium, mg	2079.5 ± 177.4	357.4 ± 154.6	1731.6 ± 205.8	483.3 ± 161.9
Chlorine, mg	1095.7 ± 136.8	202.9 ± 111.3	1333.7 ± 241.1	269.9 ± 112.6
Potassium, mg	3018.2 ± 219.3	255.4 ± 47.7	2637.3 ± 245.5	239.1 ± 63.4
Magnesium, mg	361.3 ± 35.6	36.8 ± 5.3	320.1 ± 28.1	40.9 ± 5.6
Iron mg	12.6 ± 1.1	1.4 ± 0.3	10.0 ± 1.3	1.4 ± 0.4
Zinc mg	11.2 ± 0.9	0.6 (0.2 to 1.6)	10.1 ± 1.3	0.6 (0.1 to 2.8)
Selenium, mcg	70.0 ± 11.2	0.6 (0.1 to 1.2)	59.4 ± 7.6	1.2 (0.0 to 9.0)
Copper, mg	1.5 (1.3 to 1.7)	0.1 (0.0 to 0.1)	1.1 (0.8 to 1.2)	0.0 (0.0 to 0.0)
Manganese, mg	0.6 ± 0.1	0.2 ± 0.1	0.8 ± 0.3	0.1 ± 0.1

PAs-AMP, Paralympic athletes with amputation; PAs-SCI, Paralympic athletes with a spinal cord injury; ORAC, Oxygen Radical Absorbance Capacity. No significant differences were observed between groups.

**Table 5:** Responses to the cardiopulmonary exercise test (CPET).

	PAs-AMP	PAs-SCI
Peak-RER	1.2 ± 0.1	1.2 ± 0.1
VO_2peak_, mL/kg BM/min	34.4 ± 2.9	20.6 ± 2.2 *
Peak HR, beats/min	171.5 ± 5.4	124.8 ± 14.2 *
Peak power, Watt	128.0 ± 6.1	80.0 ± 10.4 *
Peak lactate, mM	12.1 ± 1.9	6.8 ± 1.2 *
Glucose, mg/dL		
Pre-CPET	102.3 ± 3.3	105.3 ± 4.1
Difference (post–pre)	−3.0 (−12.0 to 6.0)	−12.0 (−15.5 to 1.5)
Ketones, mM		
Pre-CPET	0.1 ± 0.1	0.1 ± 0.1
Difference (post–pre)	0.3 (0.1 to 0.4)	0.2 (0.1 to 0.3)
Uric acid, mg/dL		
Pre-CPET	6.6 ± 0.5	6.1 ± 0.4
Difference (post–pre)	0.5 ± 0.3	0.3 ± 0.3
Salivary TAC, mM		
Pre-CPET	1.6 ± 0.4	1.8 ± 0.2
Difference (post–pre)	1.2 ± 0.3	0.9 ± 0.3

PAs-AMP, Paralympic athletes with amputation; PAs-SCI, Paralympic athletes with a spinal cord injury; Peak-RER, peak Respiratory Exchange Ratio at the end of the cardiopulmonary arm cranking exercise test (CPET); BM, body mass; VO_2_ peak, peak oxygen uptake; HR, heart rate; TAC, Total Antioxidant Capacity. * *p* < 0.05.

**Table 6:** Spearman’s correlations for orthorexia.

	ORTO15	ORTO7
PhA of the dominant arm	r = −0.579	r = −0.500
FM%	r = 0.570	
BMI	r = 0.569	
EnI	r = −0.542	r = −0.624
Fat %EnI	r = 0.520	
Fiber, g/d	r = −0.715	
Proteins, g/kg BM/d	r = −0.762	
NBD	r = −0.453	

ORTO15 and ORTO7 (reverse scores: high values, low orthorexia). Only significant correlations (*p* < 0.05) were reported.

## Data Availability

The data is unavailable due to privacy and ethical restrictions.

## Abbreviations

The following abbreviations are used in this manuscript:

AKA	above-knee amputation
AMP	amputation
AQUA	allergy questionnaire for athletes
AUDIT	alcohol use disorders identification
BEE	basal energy expenditure
BIA	bioelectrical impedance analysis
BKA	below-knee amputation
BM	body mass
BMI	body mass index
CPET	cardiopulmonary exercise test
EnI	energy intake
FM%:	fat mass percentage
HGS	handgrip strength
HR	heart rate
LLA	lower limb amputation
MDS	Mediterranean diet score
Med-D	Mediterranean diet
MEDScore	Mediterranean score
NBD	neurogenic bowel dysfunction
NMQ	Nordic Musculoskeletal Questionnaire
ORAC	Oxygen Radical Absorbance Capacity
ORTO	score for orthorexia
PA	Paralympic athletes
PhA	phase angle
PVA	Paralyzed Veterans of America
R	coefficient of correlation
RER	Respiratory Exchange Ratio
SCI	spinal cord injury
SSI	Starvation Symptom Inventory
TAC	Total Antioxidant Capacity
ULA	upper limb amputation
VO2 peak	peak oxygen uptake
WBAs	Wheelchair Basketball Athletes
